# Automated detection of structural alerts (chemical fragments) in (eco)toxicology

**DOI:** 10.5936/csbj.201302013

**Published:** 2013-04-06

**Authors:** Alban Lepailleur, Guillaume Poezevara, Ronan Bureau

**Affiliations:** a Normandie Univ, France; b UNICAEN, CERMN (Centre d'Etudes et de Recherche sur le Médicament de Normandie, FR CNRS INC3M - SF ICORE, Université de Caen Basse- Normandie, U.F.R. des Sciences Pharmaceutiques), F-14032 Caen, France; c UNICAEN, GREYC (Groupe de Recherche en Informatique, Image, Automatique et Instrumentation de Caen, CNRS UMR 6072, Université de Caen Basse-Normandie), F-14032 Caen, France

## Abstract

This mini-review describes the evolution of different algorithms dedicated to the automated discovery of chemical fragments associated to (eco)toxicological endpoints. These structural alerts correspond to one of the most interesting approach of *in silico* toxicology due to their direct link with specific toxicological mechanisms. A number of expert systems are already available but, since the first work in this field which considered a binomial distribution of chemical fragments between two datasets, new data miners were developed and applied with success in chemoinformatics. The frequency of a chemical fragment in a dataset is often at the core of the process for the definition of its toxicological relevance. However, recent progresses in data mining provide new insights into the automated discovery of new rules. Particularly, this review highlights the notion of Emerging Patterns that can capture contrasts between classes of data.

## Introduction

Nowadays, the understanding of the chemical risks to health and the environment represents a hot topic and *in silico* toxicology is extremely appealing because of its high throughput, its inexpensiveness, and its capacity to reduce the use of animals. By the way, according to regulators, *in silico* techniques, like Quantitative Structure-Activity Relationships (QSARs), read-across, and structural alerts have a foot in the door in the assessment of chemicals.^1–3^ The definition of structural alerts corresponds to one of the most interesting approach whose main advantage is the identification of chemicals with common mechanism of action. Indeed, investigators have always been interested in the structural and physicochemical basis of the biological behavior of chemicals. A well-known example is the Tennant and Ashby's set which defines structural alerts for DNA reactivity based on the analysis of *in vitro* mutagenicity and *in vivo* carcinogenicity data.^4^ This set of alerts has been largely superseded by others that incorporated and extended them. To date, one of the most advanced lists for evaluating the mutagenic and carcinogenic potential of chemicals is the list proposed by Benigni and Bossa,^5^ which has been implemented as a rule-based system in Toxtree^6^ and in the OECD QSAR Toolbox.^7^ Derek Nexus^8, 9^ is another example of expert system which associates structural alerts (generalized structural features in this case, like substituted vinyl ketone, 2,5-Dihalothiophene, or alkylating agent) with various toxicological endpoints (e.g. mutagenicity/carcinogenicity, skin/ocular irritation, or skin sensitization). These systems do not discover new associations, but rather store knowledge from human experts and the scientific literature, and often use a reasoning model to make a prediction. However, the expand of the knowledge base is very time consuming since it requires strong investment of domain experts and a detailed analysis of the literature. Thus, the evolution of artificial intelligence and data mining tools should benefit to the reduction of time and efforts needed to identify new structural alerts, sometimes beyond the limits of human perception. This mini-review describes different programs leading to automated detection of such information from a dataset partitioned into two data classes. These programs range from commercially available expert systems to fundamental research tools, based on algorithms that are sometimes under development.

It is impossible to make an exhaustive view of all the approaches leading to structural alerts in this mini review. We have made the difficult choice to discard the methods involving predefined fragments (chemical fingerprint) or a fix length of chemical fragments (hashed chemical fingerprint). So, some very interesting works like those carried out by Scheiber et al. (ECFP4)^10^ and Pauwels et al. (Pubchem fingerprint)^11^ are not described in this review. Two exceptions were done for the studies associated to the recent notion of Emerging Patterns (*vide infra*).

## Expert systems based on data mining approaches

### CASE,^12^ MultiCASE^13^

**Figure 1:**
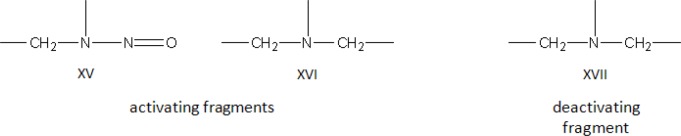
Fragments related to the rat carcinogenicity of cyclic *N*-nitrosamines according to Klopman *et al*.^12^

Historically, CASE is the first program which extracts chemical fragments from the comparative analysis of two chemical datasets. This approach identifies the most relevant descriptors, using automated algorithms, and creates expert systems capable of recognizing the existence of structural alerts in new chemicals. The program consists in tabulating, for each molecule, different fragments by breaking up the molecule into linear subunits containing between 3 and 12 interconnected heavy atoms. All fragments belonging to an active molecule are labeled active while those belonging to an inactive molecule are labeled inactive. Once all molecules have been entered, a statistical analysis of the fragment distribution is made. A binomial distribution is assumed, and each type of fragment is considered irrelevant if its distribution among actives and inactives is the same as that of the total sample of molecules. Any significant discrepancy from a random distribution of subunits between the active and inactive chemical derivatives is taken as an indication that the fragment is relevant. It is labeled as activating (biophore) if its distribution is skewed toward active molecules and inactivating (biophobe) otherwise. For example, from a training set consisting of 39 cyclic *N*-nitrosamines tested on rats (27 active carcinogens and 12 inactive compounds), two biophores and one biophobe were found by the program to have a better than 98% chance of being related to activity (Figure 1). For a new molecule, the program compares its fragments to those that are held in memory and defines a probability that the new molecule is active or not.

Concerning MultiCASE, the program is based on a hierarchical statistical analysis of a database. Like CASE, the program aims to discover chemical fragments that appear mostly in active molecules. It starts by identifying the statistically most significant fragment existing within the learning set. This first fragment is labeled as the top biophore, responsible for the activity of the largest possible number of active molecules. The active molecules containing this fragment are then removed from the database and the remaining ones are submitted to a new analysis leading to the identification of the next most significant fragment. This procedure is repeated until either the activity of all the molecules in the learning set have been accounted for or no additional statistically significant substructure can be found. For each set of molecules containing a specific biophore, MultiCASE identifies additional parameters, named modulators, which consists of the presence of certain fragments or the value of calculated parameters such as HOMO and LUMO energies, octanol–water partition coeffcient, and so on. Finally, MultiCASE proposes fragment-based QSAR models by applying a QSAR methodology for each group of molecules containing a specific biophore.

We can also mention that the same team has implemented the CASE/MultiCASE biophores in a genetic artificial neural network (GA-ANN). This new computer program was called Expert System Prediction (ESP).^14^ The purpose was to evaluate the significance of the biophores from a different point of view. The neural network learns the relationships between the patterns (represented in the form of a pattern vector) and the activities of the chemicals, and this knowledge is later used for activity prediction of new molecules. The effectiveness of the ESP approach was illustrated by studying the carcinogenicity of a diverse set of chemicals.

### PASS^15, 16^

The PASS program is based on a regression approach that provides predictions from the SAR analysis of a training set containing more than 30000 compounds. This noncongeneric database encompasses more than 500 different biological activities. The molecules were represented by “Multilevel Neighborhoods of Atoms” (MNA) descriptors,^17^ which are based on their 2D representation. Briefly, an MNA descriptors set is subdivided on levels and generated recursively. A zero-level MNA descriptor describes the atom itself and any next level MNA descriptor is the substructure notation A(D_1_D_2_...), where A is the atom A descriptor, and D_i_ is the previous level MNA descriptor of the i^th^ neighbor atom for the atom A. To estimate the activity for a new compound, its MNA descriptors have to be generated and then, the probabilities of belonging to the classes of active and inactive compounds are calculated.

### Cat-SAR^18^

**Figure 2:**
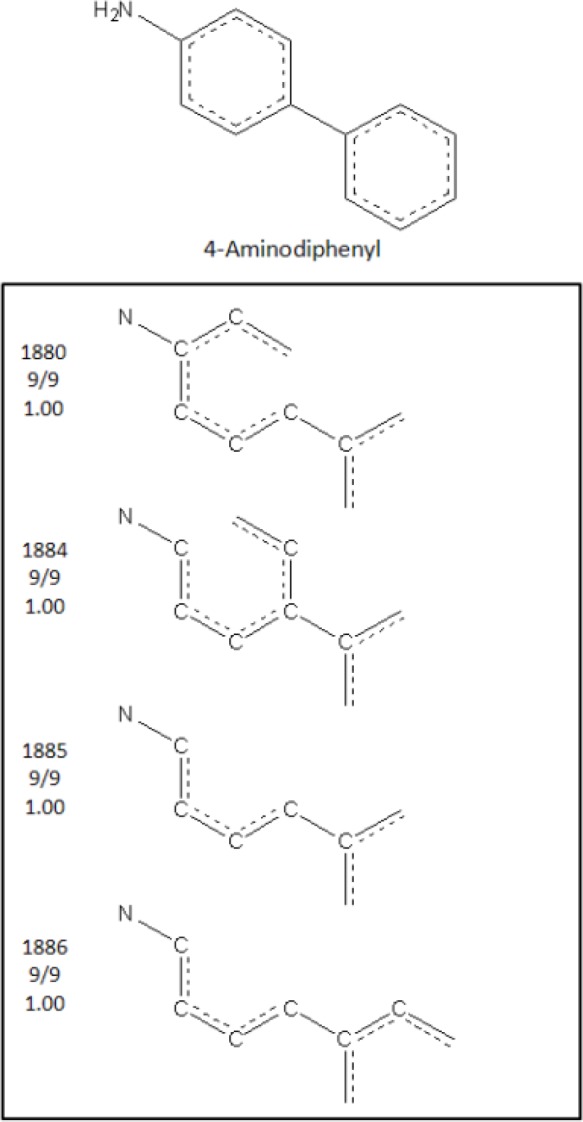
Illustration of significant fragments used to predict the carcinogenic potential of 4-aminodiphenyl adapted from Cunningham *et al*.^18^

Starting from the Tripos Sybyl HQSAR module, each chemical is fragmented into all possible substructures. HQSAR allows the user to select attributes for fragment determination including atom count, bond types, atomic connections, hydrogen atoms, chirality, and hydrogen bond donor and acceptor groups. Fragments can be linear, branched, or cyclic moieties. Models developed contained fragments between three and seven atoms and considered atoms, bond types, and atomic connections. To ascertain an association between each fragment and activity (or inactivity), the first selection rule is the number of times a fragment is identified. The second rule relates to the proportion of active or inactive compounds that contribute to each fragment. Chemical fragments are considered meaningful if they are found in at least three compounds in the learning set and are comprised of either 90% or more active or inactive compounds. To make a prediction for a new compound, the cat-SAR program determined which fragments, from the model's pool of significant fragments, the test compound contains. If none were present, no prediction of activity was made. If one or more fragments were present, the number of active and inactive compounds containing each fragment was determined. The probability of activity or inactivity was then calculated based on the total number of active and inactive compounds that went into deriving each of the fragments. For example (Figure 2), 4-aminodiphenyl was predicted to be active as a mammary carcinogen with a probability of 100%. This prediction was based on the occurrence of four similar fragments derived from nine carcinogens and zero noncarcinogens in the model's learning set. However, the example clearly highlights a redundancy issue with this program.

### LAZAR/MOLFEA^19^

**Figure 3:**
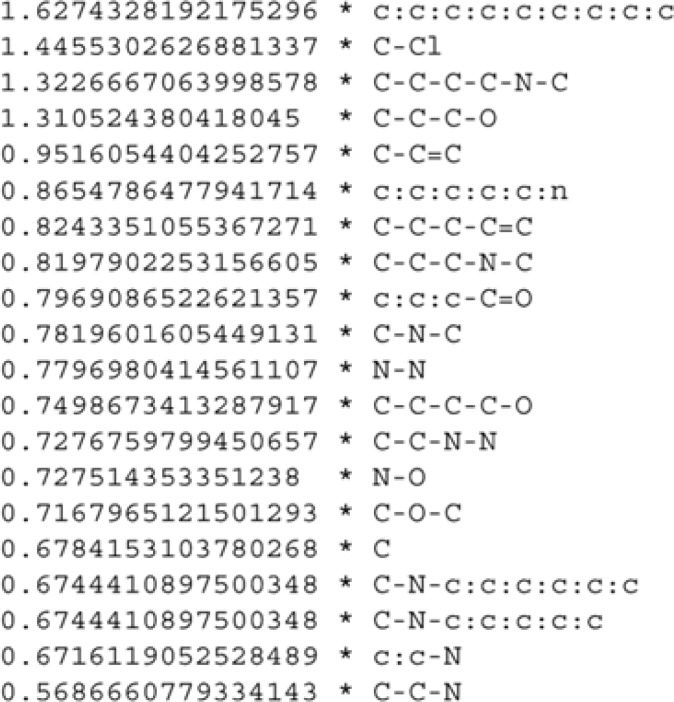
The 20 strongest activating fragments (threshold of 0.05) for Salmonella mutagenicity derived from linear SVM, and according to Helma *et al*.^19^

LAZAR, a tool for predictive toxicology, uses a simplified version of the MOLFEA algorithm, an inductive database system tailored toward discovering substructures within sets of small molecules. The considered substructures are linear molecular fragments, i.e. sequences of atoms and connecting bonds, and are based on a subset of the SMARTS language. Inductive databases are databases that can be queried not only for data but also for patterns and regularities (chemical fragments in this case) that occur within the data and fulfill a specific constraint defined by the user. For example, a frequency constraint could require that the fragment occurs in at least (respectively at most) x% of the molecules belonging to a given data set. A query in MOLFEA is composed of several conditions, each of which has to be fulfilled in order to make it a solution fragment. To efficiently compute the fragments that satisfy a given query, the generality relation on fragments is exploited. This property imposes two borders, a lower and an upper. A lower border (called the *S*-set) on the space of possible solutions contains all maximally specific fragments that satisfy the constraint. It was called a border because all fragments more general than an element of *S* will also satisfy the constraint and all fragments that are not more general than at least one fragment in *S* will not satisfy the constraint. Dually, the frequency constraint imposes also an upper border (called the *G*-set in their publication) on the space of possible solutions. To update the borders, MOLFEA applies the following algorithm. First, those fragments that do not satisfy the minimum constraint are deleted. Second, the elements of *S* are updated using a levelwise search algorithm. This algorithm keeps track of a list of candidates C_i_ and a list of solutions L_i_ to the frequency constraint. Both lists are initialized with the maximally general element T and iteratively updated. During each iteration, the candidates (fragments) associating an atom type and a bond type at level i are computed by refining existing fragments at level i-1. Those candidates satisfying the frequency threshold are retained and used to generate the candidates in the next iteration. The process is continued until no more candidates can be generated. At this point, the *S* set is computed by taking the maximally specific elements among the solutions computed that are more specific than an element of *G*. As an example, the authors identified relevant fragments (Figure 3) from a data set containing 341 mutagenic and 343 nonmutagenic compounds using the query *(freq(f, mutagens) ≥ t) Λ (freq(f, nonmutagens) ≤ t)*, where *t* varied from 0.01 (1%, 6 compounds) to 0.10 (10%, 68 compounds). Three machine learning algorithms (C4.5, PART, SVM) were used to learn SAR models from these fragments.

## Developments of graph data mining algorithms based on a frequency constraint

The tools described above have been implemented in expert system software which are generally commercial products. However, due to the evolution of the modern information methods and technology, collecting, combining, storing, and mining huge amounts of data can be done at very low costs. Indeed, several works have been developed in informatics to extract the frequent subgraphs from a dataset. Apply to the field of the chemoinformatics, those works allow to extract the frequent substructures. The frequency constraint is popular in data mining for its anti-monotonic^20^ property: if a substructure *sub* of size n (the size is the number of atoms) is not frequent in a dataset of molecular graphs, it means that all the substructure of size n+1 whose contain *sub* are not frequent in this dataset. The frequency is useful as it efficiently disregards infrequent substructures. However, as a simple remark, if according to the frequency constraint there are too few compounds that contain oxygen, then peroxide containing substructures are not explored.

Several families of algorithms exist for extracting the frequent subgraphs from a dataset of graphs^21^. The Apriori approach^22^ used a Breadth-First search strategy to cross the search space associated to the frequent subgraphs. The discovery of the frequent atoms in the dataset is the first step, then it iterates until no more frequent substructures are discovered by increasing by one the size of the candidates. At each iteration, the substructures of size n+1 are obtained thanks to the fusion of the frequent substructures of size n. A fusion process merges two frequent substructures of size n whose differ by only one atom and provides all the substructures resulting from this fusion. By testing all the possible fusions, all the candidates of size n+1 are generated. Finally, the frequency is checked and only the frequent ones are kept. The initial algorithm (named AGM) to extract the frequent subgraphs from a dataset of graphs belongs to this family. The Pattern-Growth Based approach used a Depth-First search strategy to cross the search space of the candidates. A frequent substructure of size n is extended by adding a new chemical bond leading to a candidate substructure of size n+1. Only the substructures respecting the frequency constraint are stored. The first step of this method consists to collect all the frequent atoms, then each atom are extended until no more frequent subgraphs are generated. Only substructures with at least two atoms are stored. We described here applications exploiting the Pattern-Growth Based approach.

### Gaston^23^

**Figure 4:**
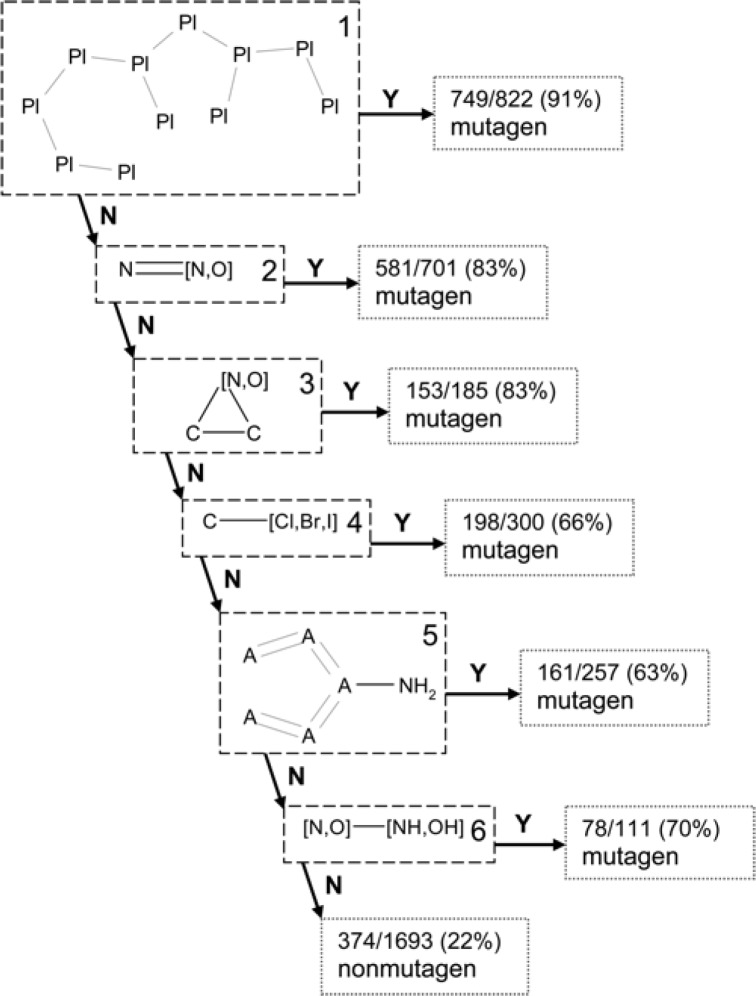
Decision list extracted from a mutagenicity data set according to Kazius *et al*.^26^

The algorithm gSpan^24^ is one of the first using the Pattern-Growth Based approach^25^ to generate the substructures. It is often used in chemoinformatics for two reasons: (i) it uses the quickstart principle: the set of the frequent substructures could be partitioning into three subsets, the subset of the paths (an atom cannot be linked to more than two other atoms), the subset of the trees (there is no cycle) and the subset of the graphs whose contain cycles and (ii) the authors make available its source code. For an illustration of the potential of Gaston, an elaborate method of graph-based chemical representation was developed by Kazius *el al*.,^26^ and tested for extracting substructures from a mutagenicity data set (4337 entries). At the start of this process, the initial substructure is mapped everywhere it fits into every molecule in the data set. For each such mapping, the atoms at the neighboring positions of this substructure are stored. For each substructure in such a collection, its statistical association with mutagenicity, expressed as the p-value, was determined from the amounts of mutagens and nonmutagens. It was then determined which substructure was most strongly associated with mutagenicity, that is, which substructure possessed the lowest p-value. This substructure was then selected to split the chemical data set into two subsets (linear decision trees). Each split generated one subset of compounds that contain this substructure and another subset of compounds that lack this substructure. This latter subset was used to recompute the p-values of all substructures. From these p-values, the next most mutagenic substructure was determined and then used to split this chemical subset in two, and so on. After six splits, all compounds from the original database are divided over seven subsets. The result is illustrated in Figure 4. The statistics of mutagenicity prediction based on 10 fold cross-validation showed a sensitivity of 83% and a specificity of 74%.

### MOSS/MoFa^27^

**Figure 5:**
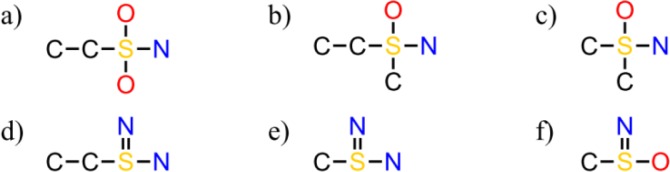
Set of six example molecules.

**Figure 6:**
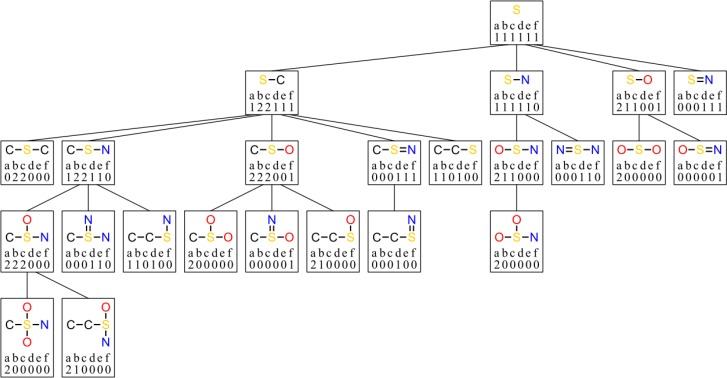
Search tree from the six previous molecules.

**Figure 7:**
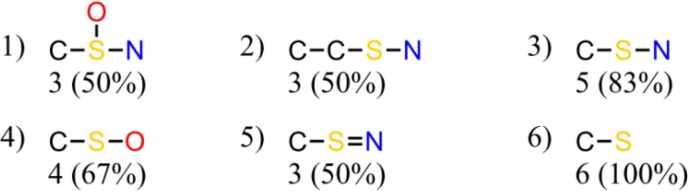
Frequent fragments found from the example of Borgelt *et al*.^27^

Concerning this program, the association rules start from an algorithm similar to Eclat^28^ (search trees). The process is well described in the publication and is illustrated on a set of molecules (Figure 5) with a minimum support (frequency) of 50%. First the sulfur atom is embedded forming the root of the search tree (Figure 6), and then the embeddings are extended in all possible ways. Of course, subtrees of the search tree are pruned if they refer to substructures not having enough support. This leads to the definition of six frequent substructures starting from the example molecules (Figure 7). A crucial step concerns the definition of the starting point (sulfur in this example). We can start with a different atom, as long as this atom is rare in the molecule, or a specific core like an aromatic ring with one or two side chains for instance. Contrast structures are then extracted corresponding to substructures that are frequent in a predefined subset of the molecules and infrequent in the complement of this subset. Experimental results concerned HIV-1 infection and they extracted nitrogen based, sulfur-based, and selenium based fragments particularly.

## From frequent patterns to emerging patterns

In informatics, the recent studies of pattern mining have given more attention to the discovery of patterns that are “significant”, “emerging”, “dominant” and so forth, than simply frequent. Indeed, it appears that frequent patterns alone have a limited applicability and usability in terms of building predictive models.^29^ Thus, methods of finding representative subsets of frequent patterns that could be effectively useful are appealing. In this part, we will focus on the notion of Emerging Patterns and its applications in the field of chemoinformatics.

### Emerging patterns^30^

Emerging Patterns (EPs) were introduced by Dong and Li. The emerging constraint captures differentiating characteristics between two classes of data. An EP is defined as an itemset which support (i.e. its number of occurrences in the dataset) increases significantly from one dataset D_1_ to another D_2_. Dong and Li have proposed to use the growth rate measure to evaluate this increasing. The growth rate of a pattern *pat* from D_2_ to D_1_ is given by the ratio of the frequency of *pat* in D_1_ over the frequency of *pat* in D_2_. In this way, EPs capture contrasts between two data classes. An interesting point on this project is to discover EPs with small support. The authors precise that it is a challenge due to two reasons: i) the useful anti-monotonic property no longer holds for EPs, and ii) there are usually too many candidates. Naïve algorithms, which consider all itemsets, are not viable since it would be too costly. However, such collections of itemsets have a nice property corresponding to the notion of closed intervals.^31^ If X and Z are in S and Y is a set such that X ⊆ Y ⊆ Z then Y is in S. Thus, they described large interval-closed collections of itemsets using borders, defined as the pair of the sets of the minimal and the maximal itemsets. Clearly, borders are usually much smaller than the collections they represent. Such borders can be efficiently discovered by algorithms like Max-miner.^32^ For instance, on a mushroom dataset with a growth rate threshold of 2.5, 2^28^ EPs are possible but they can be represented by only half a million borders. Otherwise, if the support of an EP in D_2_ is null then this pattern was called a Jumping Emerging Pattern (JEP). A JEP is defined as the most expressive EP.^33^

**Table 1:** Coverage rate of the JEFs on the H400 molecules of the learning set, and success rate of the prediction rule on the testing set.

		Frequency threshold (%)					

		5	4.3	3	2.6	1	0.6
Learning set	Support in H400 molecules	15	13	9	8	3	2
	Coverage rate on H400 (%)	34.3	41.5	60.4	62.9	81	84.3
	Coverage rate on H400 (dev)	6.43	4.9	3.83	2.7	1.27	0.74
Testing set	H400 success rate (%)	38.3	42.9	62.6	66.9	79	81.9
	H402 success rate (%)	95.8	94.3	85	81	55.8	47.1
	Overall success rate (%)	58.1	60.6	69.9	70.7	71	69.9

### Emerging Chemical Patterns^34^

In 2006, Auer and Bajorath applied for the first time the concept of EPs in chemoinformatics. They introduced the notion of Emerging Chemical Patterns (ECP) as a novel approach to molecular classification. The authors used the subset of the JEP to conduct an experimental study. An hypergraph-based algorithm was applied to mine the JEPs from two classes of data, actives or inactives. The potential of this approach to classify derivatives was analyzed on four publicly available compound data sets. But in this case, they do not use molecular graphs, they used a set of sixty one 1D and 2D molecular descriptors with values ranges discretized into suitable intervals. On the basis of their results, ECPs are expected to broaden the spectrum of molecular classification methods, and complement computational methodologies like binary QSAR and decision trees.

### Jumping Emerging Fragments^35^

This study corresponds to the first application of EPs to a classification task in ecotoxicology. The authors assumed that the level of toxicity for a chemical may be influenced by the presence of a specific molecular fragment. Such a fragment has been called a Jumping Emerging Fragment (JEFs) since it has a strong foothold in the toxic chemicals and is missing from the non-toxic chemicals. A three-step algorithm that automatically extracts chemical fragments was designed. Let D be partitioned into two subsets D_1_ and D_2_. The first step is to extract the frequent connected subgraphs in D_1_ according to the frequency threshold. For this step, a Pattern-Growth Based algorithm was used instead of an Apriori Based algorithms.^36^ According to an experimental comparison of four Pattern-Growth Based algorithms,^37^ gSpan^24^ was chosen for a question of memory and speed. The second step consists in defining for each graph G_D_ of D and for each connected graph G resulting from step 1, if G is a subgraph of G_D_. For that task, an in-house implementation of the J.R. Ullmann's algorithm^38^ was used to solve the resulting multiple subgraph isomorphism problems. For the third step, the problem is described by items (presence or absence of each frequent connected graph) and Music-DFS algorithm^39^ was used to discover JEFs. The authors applied this methodology to discover JEFs from H402 (harmful to aquatic life) to H400 (very toxic to aquatic life) chemicals, and parametrize the simplest possible decision rule based on these jumping fragments: a molecule is H400 just in case it contains a JEF. The analysis of the results obtained on the testing set, in function of the frequency and the coverage rate on H400 molecules (learning set) has shown that a JEF recorded at a high frequency threshold is meaningful to define the toxicity of a derivative (see the H402 success rate for 3-5%).

### Representative Pruned Molecular Patterns^40^

In comparison with previous study, the authors consider now the conjunction of fragments, i.e. the combination of different moieties of a molecule in only one pattern. The new notion of Representative Pruned Molecular Patterns (RPMPs) was introduced. As a reminder, when a dataset is partitioned into targeted examples and non-targeted ones (also called “classes”), the growth-rate of a pattern is defined as the ratio between its frequency in the target class over its frequency outside the target class. The first step consists in the enumeration of all the frequent and emerging molecular patterns (FEMPs) according to frequency and growth rate constraints. In practice, FEMPs are often numerous and include redundant information, but they could be condensed by applying the notion of closed pattern.^41^ A closed pattern is a pattern for which no element can be added without decreasing its extent, i.e. the set of molecules in which the molecular pattern occurs. Thus, by retaining only closed FEMPs the authors can condense the important set of FEMPs without losing information. However, closed patterns tend to be very long (number of fragments) since a large part corresponds to subfragments of a larger fragment. These redundant subfragments can be pruned without losing information. These resulting shorter representations have been called Representative Pruned Molecular Patterns (RPMPs). The illustrative example corresponds to a dataset of 295 chemicals annotated by their toxicity to aquatic life (223 toxic and 172 non-toxic chemicals). The Table 2 indicates the evolution of the number and length of the corresponding patterns in function of the growth rate values (minimum frequency threshold of 2.8%).

**Table 2:** FEMP *vs* RPMP in function of the growth rate.

Growth rate	Number of patterns			Length of patterns	

	FEMP	Closed FEMP	RPMP	Closed FEMP	RPMP
2	2 414 271 394	735	735	15.2	2.29
5	1 629 688 309	415	415	15.7	2.36
10	1 629 688 309	264	264	16.4	2.4
25	1 632 132 495	238	238	16.9	2.43
∞	1 632 131 769	236	236	16.9	2.43

**Figure 8:**
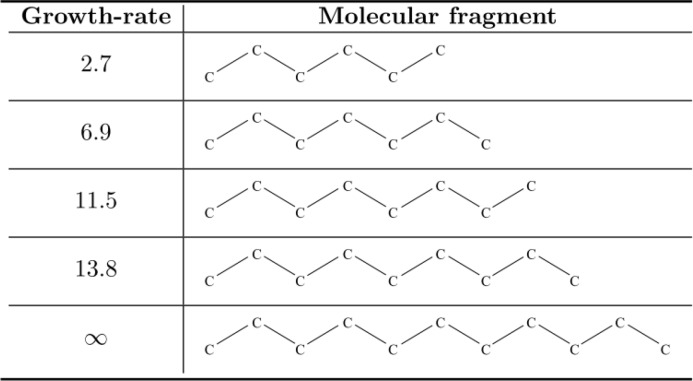
Growth-rate values of the alkyl chains according to their length.

Besides, the interpretability of the RPMPs seems to be more obvious. A simple illustration deals with the impact of the length of an alkyl chain towards the ecotoxicity. The RPMPs show an evident relationship between the growth-rate values and the number of carbon atoms of the fragments (see Figure 8). The meaningful length of the alkyl chains begins for C6 (growth-rate of 2.7), it increases strongly for C7 (growth-rate of 6.9), to reach a maximum value for C11 (growth-rate of 8, corresponding to a Jumping Emerging Pattern). Thus, in terms of structure-activity relationships, this result highlights that the hydrophobicity of an alkyl chain is correlated with its length, and is in straight relation with its ecotoxic effect. Several other examples are given in the article.

### Jumping Emerging Patterns^42^

**Figure 9:**
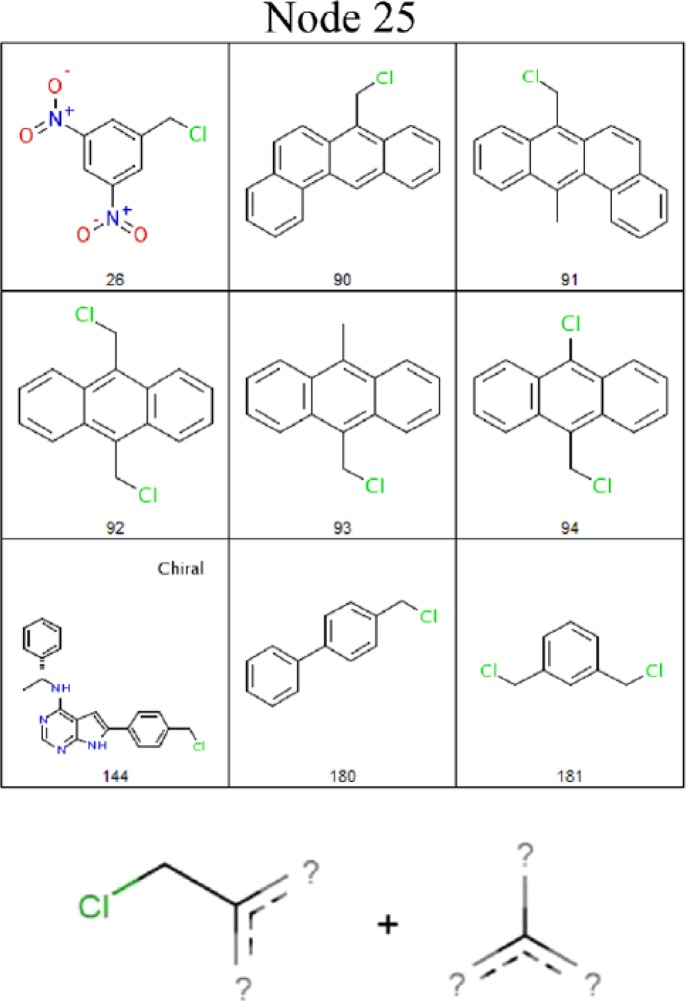
Example of JEPs (bottom) for mutagenic alkylating agents and supporting active compounds (top), according to Sherod *et al*.^42^

Recently, researchers of the Sheffield University collaborated with Derek Nexus developers to help automate the process of knowledge extraction from toxicity data sets. Their approach is based on the discovery of Jumping Emerging Patterns (JEPs). In this work, structural fingerprints are used as descriptors. The complete procedure for mining the JEPs is in 6 steps: i) generation of all atom pairs under user-defined constraints from the active compounds in the data set, ii) application of the Horizon-Miner algorithm to extract the maximal patterns for both the actives and the inactives, iii) application of the border-differential algorithm to mine the set of all possible minimal JEPs, iv) reduction of the set of minimal JEPs to those that occur in distinct sets of actives, v) identification of the relationships between the supporting actives of minimal JEPs and arrangement of them into hierarchies, and vi) extraction of the maximum set of commonly occurring atom pairs from the set of actives that support each minimal JEP. The illustrative examples deal with Ames mutagenicity, oestrogenicity, and hERG channel inhibition end points. The method is effective to cluster the data sets around minimal jumping-emerging structural patterns and finding descriptions of potentially activating structural features. Furthermore, the mined structural features have been shown to be related to some of the known alerts for all three tested end points. For example, Figure 9 highlights a JEP for well-known mutagenic alkylating agents.

## Conclusion

The characterization of a chemical fragment or a chemical pattern associated to a toxicological profile is of first interest by considering the continuous development of chemoinformatics tools around this topic. Owing to the fact that a single chemical fragment is not always responsible for the overall toxicity of a chemical, the present objective is to analyze the combination of chemical fragments (chemical patterns) leading to an increase or a decrease of toxicity starting from a referential toxic fragment. The first described approach corresponds to the Klopmann's method (CASE/MultiCASE) which extracts meaningful chemical fragments (named biophores) in function of their distribution between two datasets. Afterwards, these biophores were associated to QSARs or implemented in classification tools, leading to the first commercialized expert systems. Concerning the extraction of chemical fragments, a second generation of tools was more recently developed, corresponding to the Pattern Growth-Based algorithms (like Gaston). They led to very interesting results in terms of characterization of the fragments and statistical results for the estimation of the toxicity. The last evolution corresponds to the search of representative subsets of frequent patterns. In this review, we emphasize the notion of Emerging Patterns (EP), whose extraction is based on the notion of contrast (growth rate) between two datasets. We are at the beginning for EPs but, their potential in terms of statistics and interpretability related to the toxicological profile of chemical derivatives is really promising.^43^ The size of the EP set (number of patterns) in function of the size of the initial dataset is really an issue, and the notion of border does not seem to be sufficient to solve it. As described in the review, Representative Pruned Molecular Patterns (RPMPs) represents a first way to reduce this size without losing chemical information but to go further, an analysis of the relationships between the RPMPs must be carried out. This is underway.
